# Evaluation of CHROMagar™ *B. cepacia* agar for the detection of *Burkholderia cepacia* complex species from sputum samples of patients with cystic fibrosis

**DOI:** 10.1007/s10096-024-04845-4

**Published:** 2024-05-23

**Authors:** Ainhize Maruri-Aransolo, Juan de Dios Caballero, Malkoa Michelena, María José Medina-Pascual, Gema Carrasco, Oscar Asensio, Maria Cols, Rafael Cantón, the GEIFQ group

**Affiliations:** 1 Servicio de Microbiología, Hospital Universitario Ramón y Cajal and Instituto Ramón y Cajal de Investigación Sanitaria (IRYCIS), Madrid, Spain; 2 CIBER de Enfermedades Infecciosas CIBERINFEC, Instituto de Salud Carlos III, Madrid, Spain; 3 Laboratorio de Referencia e Investigación en Taxonomía, Centro Nacional de Microbiología, Instituto de Salud Carlos III, Majadahonda, Madrid, Spain; 4 Pediatric Pulmonology Unit, Consorci Corporació Sanitària Parc Taulí, Sabadell, Spain; 5 Pediatric Pulmonology Department and Cystic Fibrosis Unit, Hospital Sant Joan de Déu, Barcelona, Spain

**Keywords:** CHROMagar™, *Burkholderia cepacia* complex, Cystic fibrosis, Chronic respiratory infections

## Abstract

**Introduction:**

*Burkholderia cepacia* complex (BCC) are non-fermenting Gram-negative bacteria that can chronically colonize the lungs of people with cystic fibrosis (pwCF), causing a severe and progressive respiratory failure, post-transplant complications and epidemic outbreaks. Therefore, rapid and accurate identification of these bacteria is relevant for pwCF, in order to facilitate early eradication and prevent chronic colonization. However, BCCs are often quite difficult to detect on culture media as they have a slow growth rate and can be hidden by other fast-growing microorganisms, including *Pseudomonas aeruginosa* and filamentous fungi.

**Material and methods:**

We evaluated the sensitivity of CHROMagar™ *B. cepacia* agar using 11 isolates from a well-characterized BCC collection, using BCA agar (Oxoid, UK) as a gold standard. We also studied 180 clinical sputum samples to calculate positive (PPV) and negative (NPV) predictive values. Furthermore, we used three of the well-characterized BCC isolates to determine the limit of detection (LOD).

**Results:**

Eleven isolates grew on CHROMagar™ *B. cepacia* at 37ºC after 48 h. The NPV and PPV of CHROMagar™ *B. cepacia* were 100% and 87.5%, respectively. The LOD of CHROMagar™ *B. cepacia* was around 1 × 10^3^ CFU/ml, requiring a ten-fold dilution lower bacterial load than BCA for BCC detection.

**Conclusion:**

CHROMagar™ *B. cepacia* agar proved to have a very good sensitivity and specificity for the detection of clinical BCCs. Moreover, the chromogenic nature of the medium allowed us to clearly differentiate BCC from other Gram-negative species, filamentous fungi and yeasts, thereby facilitating the identification of contaminants.

**Supplementary Information:**

The online version contains supplementary material available at 10.1007/s10096-024-04845-4.

## Introduction

In people with cystic fibrosis (pwCF), chronic bacterial colonization of the respiratory tract by opportunistic pathogens is a leading cause of morbidity and mortality [1]. Pathogenic bacteria such as *Staphylococcus aureus* or *Pseudomonas aeruginosa* mainly cause infections, but nonfermenting Gram-negative bacilli such as *Stenotrophomonas maltophilia, Achromobacter xylosoxidans* or *Burkholderia cepacia* complex (BCC) are increasingly found [2]. BCC is a group that includes more than 22 species that are closely related to each other phenotypically, but genetically distinct [3]. Until not many years ago, BCC included nine species: *B. cepacia*, *B. multivorans*, *B. cenocepacia*, *B. stabilis*, *B. vietnamiensis*, *B. dolosa*, *B. ambifaria*, *B. anthina* and *B. pyrrocinia*. However, this number has increased in recent years, including in the complex other species such as *B. ubonensis*, *B. latens*, *B. diffusa*, *B. arboris*, *B. seminalis*, *B. metallica*, *B. contaminans* and *B. lata* [4–6]. These bacteria are ubiquitous in the environment and can survive long periods in hostile environments such as disinfectants, distilled water, and nebulizers [1, 7]. In Spain, the two most clinically prevalent species in pwCF were traditionally *B. cenocepacia* and *B. multivorans* but their prevalence has declined in recent years and there has been an increase in *B. contaminans* infections [8, 9]. BCC infections are generally opportunistic and are treated with ceftazidime and other extended spectrum cephalosporins due to their intrinsic resistance to other antimicrobials [10]. In pwCF, however, this complex is among one of the most important pathogens since it can cause the so-called "*B. cepacia* syndrome", a severe and progressive respiratory failure with bacteremia, very difficult to eradicate, that causes a decrease in life expectancy of patients [11]. Some studies have shown that BCC bacteria are the pathogens that have the most negative impact on the patient's lung function, only surpassed by *Mycobacterium abscessus* in severity [12]. Therefore, a rapid and accurate identification of these bacteria is relevant for pwCF.

**Fig. 1:**
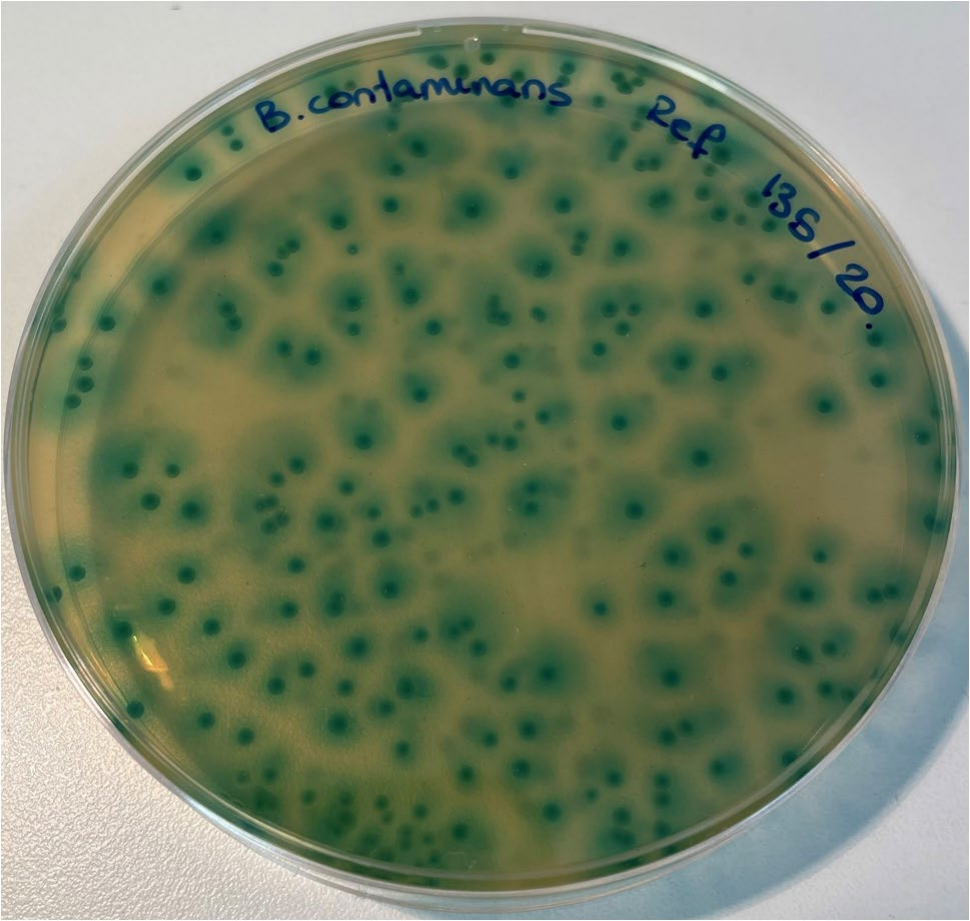
Growth of *Burkholderia contaminans* in CHROMagar™ *B. cepacia* agar. Typical *Burkholderia cepacia* complex colonies growth as blue colonies with a surrounding halo

Isolates from BBC grow slowly in normal culture media and could go unnoticed in pwCF due to the massive growth of other organisms in respiratory samples unless selective media are used [13]. Different commercial culture media have been marketed for the detection and identification of BCC isolates. CHROMagar™ *B. cepacia* (CHROMagar Paris, France) is a new, not yet commercialized, highly selective chromogenic medium for the detection of most BCC bacteria. This selective medium limit or inhibits the growth of yeasts, filamentous fungi, unwanted Gram-negative bacteria and completely inhibits the growth of Gram-positive bacteria. BCC strains should grow as blue colonies with a surrounding halo, mostly at 36 h, but growth may be delayed until 48 h (Fig. 1).

In this article we evaluate the sensitivity of the CHROMagar™ *B. cepacia* medium using: i) pure isolates from a well-characterized BCC collection (March 2013 – November 2013) [9] and ii) clinical samples to determine the positive and negative predictive values iii) three isolates to determine the limit of detection (LOD) [9].

## Materials and methods

To assess the sensitivity of the medium, 11 BCC isolates previously characterized in our laboratory were used. These isolates were obtained from pwCF during a multicenter study (2013) conducted at national level in Spain [9]. A 0.5 McFarland suspension of these strains was prepared using fresh cultures grown overnight on Columbia blood agar at 37℃. Fifty microliters of these suspensions were seeded on CHROMagar™ *B. cepacia* and incubated at 37℃ for 48 h*.*

For the second part of the study, a total of 180 sputum samples were analyzed. Seventy-three pwCF who attended at our center (Ramón y Cajal University Hospital) provided fresh samples. The rest of them (*n* = 107) came from pwCF included in a national multicenter study (January 2021 – November 2021; 6 CF Units from 6 Spanish Hospitals) and were kept frozen at -80℃ until processing at our center. The samples were slowly thawed at 4 °C (when necessary) and cultured following standardized protocols (see Supplementary material) [9]. For BCC isolation, samples were cultured on CHROMagar™ *B. cepacia* and, in parallel, on *Burkholderia cepacia* medium (BCA, OXOID, UK; product ref: PO0938A). BCA is a pre-poured plate which is routinely used in our clinical microbiology laboratory to detect BCC. CHROMagar™ *B. cepacia* is a dehydrated culture medium that was prepared following manufacturer’s instructions. Both media were incubated for 48 h at 37 °C, following an extended incubation at room temperature for a further 7 days [9]. All isolates that grew on both media were identified using MALDI-TOF MS (Bruker-Daltonics, Germany) [14]. The growth of other microorganisms than BCC on both media was also recorded.

To carry out the third part of the study, LOD was assessed using CHROMagar™ *B. cepacia* medium and BCA as well as 3 clinical isolates (2 *B. cepacia*, 1 *B. vietnamiensis*) [15]. The isolates were suspended in NaCl 0.9% to a density equivalent to 0.5 McFarland (*ca.* 2 × 10^8^ CFU/mL), serial tenfold dilutions were performed and 100 µL were plated on CHROMagar™ *B.cepacia* medium and on BCA medium and incubated at 37ºC for 48 h to count viable colonies. The experiments were performed in triplicate.

## Results

For the first target, we seeded the 11 well-characterized BCC strains from a previous multicenter study and found that 11/11 (100%) isolates grew after 48 h at 37 °C. Four of the 11 isolates grew well at 36 h, but most needed up to 48 h for good growth.

Regarding the second objective, the growth data on both media used to calculate the negative and positive predictive values are shown in Table 1. Twelve out of the 180 sputum samples were positive for BCC isolates on BCA medium and 14 samples were positive on CHROMagar™ *B. cepacia* medium (two additional isolates). It should be noted that the number of colonies growing on CHROMagar™ *B. cepacia* plates from these two samples was very small, being 18 CFU/ml for one and 27 CFU/ml for the other.

**Table 1:** Growth on CHROMagar™ *B.cepacia* and on BCA media

Microorganisms present in sputum samples	Growth in:		
	Overall^a^	CHROMagar *B. cepacia* medium	BCA medium
BCC	14	14 (100%)	12 (85.7%)
Gram—^b^	74	13 (17.5%)	7 (9.4%)
*Serratia marcescens*	2	2 (100%)	1 (50%)
*Achromobacter xylosoxidans*	19	11 (57.8%)	5 (26.3%)
*Pseudomonas aeruginosa*	31	0	1 (3%)
*Klebsiella* spp*.*	5	0	0
*Enterobacter* spp.	8	0	0
*Escherichia coli*	3	0	0
Others	6	0	0
Gram +	124	0	0
Yeast	55	20 (36.36%)	19 (34.5%)
Filamentous fungi	44	6 (13.6%)	13(29.5%)

^a^Overall number considering growth on either medium

^b^ Not counting BCC isolates

Different filamentous fungi, yeasts and Gram-negative bacilli that were not BCC grew in both media, whereas all Gram-positives were inhibited. Among Gram-negative bacteria, *Achromobacter xylosoxidans* was the most frequently not inhibited on either medium (42.2% and 73.7% of inhibition on CHROMagar™ *B. cepacia* and BCA, respectively). We also observed in two occasions the growth of a multiresistant *Serratia marcescens* in both media. Thirty-one samples were positive for *Pseudomonas aeruginosa*, but this microorganism grew only in one case on BCA medium and not on CHROMagar™ *B. cepacia*. To note that a relatively high percentage of yeasts were able to growth in both media and, regarding filamentous fungi, only half of them were able to grew on CHROMagar™ *B. cepacia* medium.

Considering as positive any kind of growth on both media, negative predictive values (NPV) and positive predictive values (PPV) of CHROMagar™ *B. cepacia* medium were 100% and 20.9%, respectively. The corresponding values for BCA were 98% and 19%, respectively. The PPVs were low in both media because there was growth of other microorganisms. Nevertheless, in the CHROMagar™ *B. cepacia* medium colonies that were not BCC could be easily identified by their white (not blue) color except for two *A. xylosoxidans*, whose colonies had a blue halo. In fact, we must consider that both media are chromogenic so we reanalyzed the PPV and the NPV considering the bluish (CHROMagar™ *B. cepacia*) or the pinkish (BCA) color of the colonies. The new NPV and PPV obtained for CHROMagar™ *B. cepacia* were 100% and 87.5%, respectively, and the corresponding ones for BCA were 95.9% and 75%, respectively.

The limit of detection (LOD) of BCC on CHROMagar™ *B. cepacia* and BCA media were determined using 3 isolates. The media LOD varied. In the case of CHROMagar™ *B. cepacia*, the LOD were around 1 × 10^3^ CFU/ml, but in the case of BCA medium it was 1 × 10^4^ CFU/ml. Therefore, to detect BCCs on BCA medium, we need a ten-fold higher bacterial load of the organism in a sputum sample (see Table 2).

**Table 2:** LOD for the three clinical strains (RYCFIS19B01, RYCFIS19B02, RYCFIS19B03)

	LOD (CFU/ml) ^a^	
Microorganism	CHROMagar™ *B. cepacia*	BCA
*B. vietnamiensis* RYCFIS19B01	5.93E + 03	3.88E + 04
*B. cepacia* RYCFIS19B02	3.83E + 02	8.28E + 03
*B. cepacia* RYCFIS19B03	4.75E + 03	6.65E + 04

^a^Mean value of three counts

## Discussion

This is the first study to test the accuracy of CHROMagar™ *B. cepacia* medium in the detection of BCC isolates and its ability to inhibit other microorganisms. There are few studies comparing different culture media to detect BCC from clinical sputum samples from pwCF. Marrs et al. (2021) compared 3 culture media: *Burkholderia cepacia* selective agar (BCSA, bioMérieux), BD Cepacia medium (Becton–Dickinson) and MAST Cepacia medium (MAST Diagnostics), the sensitivity of BCSA being the best of all media (100%) [16]. In our study, CHROMagar™ *B. cepacia* medium had also 100% sensitivity, being comparable to that of the BCSA. It should be noted that we did not obtain any false negative results. Among false positive growths, most of them were easily discriminable since it did not present the characteristic blue halo. Only 2 *A. xylosoxidans* grew with a blue halo, reducing the PPV to 87.5%. The concordance that we obtained with both media in the PPV and NPV values was very similar. The main advantage of the evaluated medium lies in its specific selective properties, which facilitate the direct detection of BCC isolates due to the blue halo they present in the medium with the absence of growth of non-BCC isolates. This advantage has been observed for other different chromogenic media that have shown similar good sensitivity and specificity results in detecting pathogenic or multiresistant bacteria [17, 18]. We should point out that, despite the positive results in the evaluation of CHROMagar™ *B. cepacia*, additional MALDI-TOF screening should be still needed to confirm results.

Sputum samples also contain bacteria commonly found in hospitals, such as different Enterobacterales or *Staphylococcus aureus*. Interestingly, the CHROMagar™ *B. cepacia* medium was able to inhibit the growth of all Gram-positives and most Enterobacterales. This is important since other fast-growing bacteria can delay the detection of BCC by overgrowth when using standard culture media. Therefore, having a high specificity can be an advantage in the context of CF, where slow-growing pathogens can easily be obscured by other bacteria or fungi [19]. Regarding Enterobacterales, only 2 isolates of multiresistant *S. marcescens* were able to grow on CHROMagar™ *B. cepacia*, probably due to its intrinsic resistance to colistin. With regard to *A. xylosoxidans*, it grew more often on CHROMagar™ *B. cepacia* medium than on BCA medium. However, the absence of a characteristic blue halo allowed us to differentiate it properly from BCC in all but two cases. Moreover, *A. xylosoxidans* is an emerging CF pathogen which has also a fastidious growth, so the possibility to isolate it on CHROMagar™ *B. cepacia* agar could be considered an advantage.

Concerning the molecular methods, specific PCR on direct sample has demonstrated higher sensitivity and specificity than culture media in some studies [19, 20]. This is probably due to a low bacterial load in sputum samples. In our experience, we saw a relatively low LOD of CHROMagar™ *B. cepacia* medium (1 × 10^3^ CFU/mL), so maybe it should be advisable to seed a larger sample volume to enhance the sensitivity.

A drawback of CHROMagar™ *B. cepacia* medium is that it must be manually prepared, and this can be somewhat laborious and time-consuming to be performed in clinical laboratories. Another limitation of our work is the low number of BCC positive samples studied, which can be explained by the low prevalence of these microorganisms in our pwCF [21].

In summary, we have evaluated the efficacy of CHROMagar™ *B. cepacia* medium and has shown a very good sensitivity and specificity for the detection of BCC. In addition, this chromogenic medium allows to differentiate the growth of other species in a way that is easy to interpret. This feature may allow its incorporation into clinical microbiology laboratories.

### Supplementary Information

Below is the link to the electronic supplementary material.

Supplementary file1 (DOCX 457 KB)
