# Supplementary material for: Evaluation of CHROMagar™ B. cepacia agar for the detection of Burkholderia cepacia complex species from sputum samples of patients with cystic fibrosis
Source: Eur J Clin Microbiol Infect Dis. 2024 May 23;43(7):1349–53. doi: 10.1007/s10096-024-04845-4 (PMC11271321; doi:10.1007/s10096-024-04845-4)
Supplement: Supplementary file 1 — Supplementary file1 (DOCX 457 KB) [file 10096_2024_4845_MOESM1_ESM.docx]

**S1. Sputum seeding procedure used in our center.**


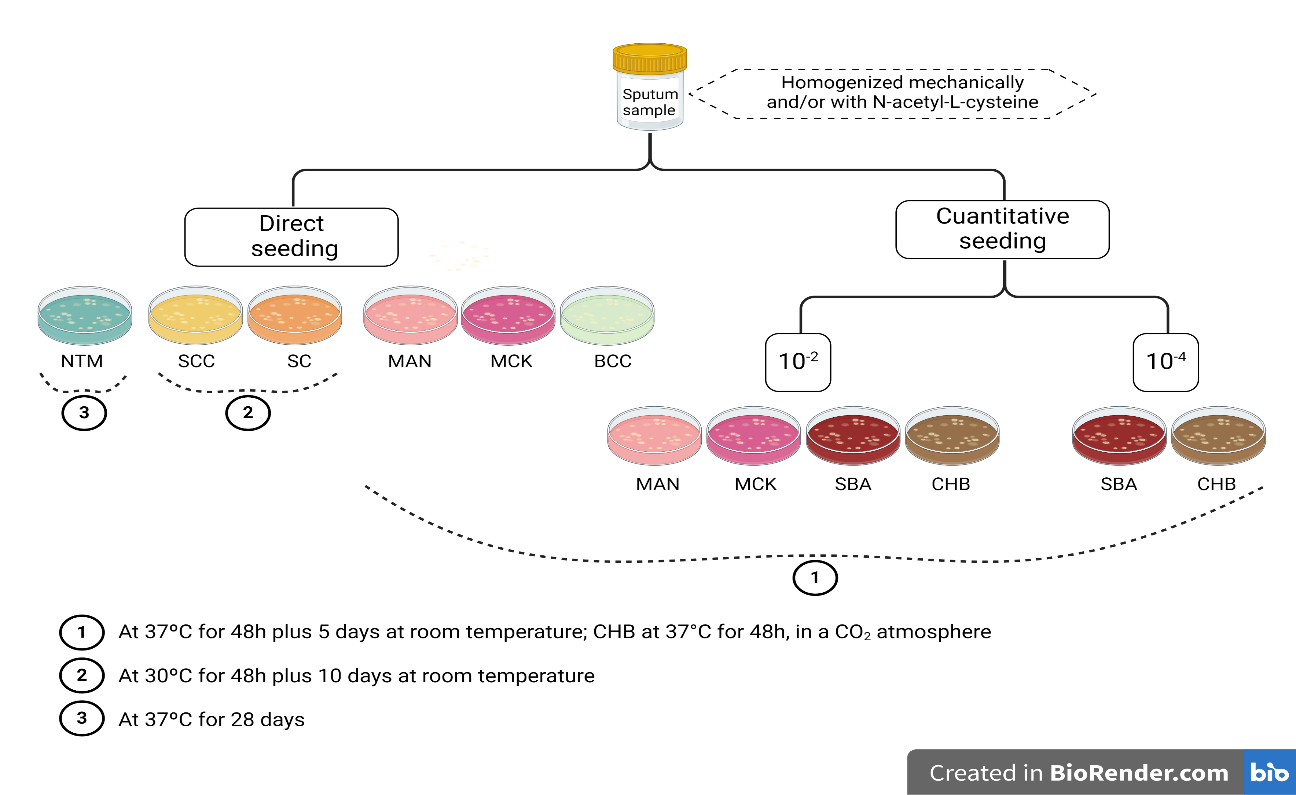


*

**

1. At 37ºC for 48h plus 5 days at room temperature; CHB at 37ºC for 48h, in a CO_2_ atmosphere.
2. At 30ºC for 48h plus 10 days at room temperature.
3. At 37ºC for 28 days.

* 50 µL of direct sample in each culture medium.

** Serial dilutions are performed with 50 µL of the sample in 4.95 mL of sterile saline solution (10^-2^). The 10^-4^ dilution is prepared with 50 µL of the 10^-2^ dilution in 4.95 mL of sterile saline solution. Finally, 50 µL of each dilution were used to inoculate each corresponding plate.

NTM: Non-tuberculous mycobacteria agar; SC: Sabouraud dextrose agar with chloramphenicol; SCC: Sabouraud dextrose agar with chloramphenicol and cycloheximide; Man: Mannitol agar; MCK: MacConkey agar; BCC: *Burkholderia cepacia* Selective agar; SBA: Columbia-agar with sheep-blood; CHB: Columbia-agar with chocolate horse-bood plus bacitracin. Created with BioRender.com.

**S2. Calculation of the number of colonies per milliliter**

To calculate the CFU/mL in the original sample, the following formula was used:

$$N=n*\frac{\mathrm{Vt}}{\mathrm{Vs}}*20*D$$

N: total number of CFU/mL.

n: number of colonies counted on a specific culture medium.

Vt: total volume, corresponding to the initial volume of the sample (Vs) + volume of N-acetylcysteine required for homogenization.

20: Correction factor associated with seeding of 50 µL per plate (the count is reported per mL of sample).

D: Dilution factor. Correction factor associated with dilutions 1/1; 1/100 and 1/10,000 for quantitative culture.
